# Supplementary material for: Maintaining essential healthcare services in Addis Ababa during COVID-19: A qualitative study
Source: PLoS One. 2024 Dec 27;19(12):e0308534. doi: 10.1371/journal.pone.0308534 (PMC11676944; doi:10.1371/journal.pone.0308534)
Supplement: S1 File — (DOCX) [file pone.0308534.s001.docx]

**Health facility Key informant interview guide (ERP/focal person for sustaining essential health service)**

Code No. _________________________

Age_______

Sex____________

Occupation____________________

Date of Interview____________

Place of Interview__________________________________________

Time of Interview__________________________________________

1. Can you tell me about the effect of COVID-19 on the provision of essential health care services? (With time comparison)

Probe

- Which service is most affected? Why?
- On community health care seeking
- On health workers
- On supply/ resource including essential medications stock out
- On PPE

1. Can you tell me about the process/ activities undertaken/ established for sustaining essential health care services?

Probe to see:

- Interventions like
  - How they prioritized essential health service
  - Telemedicine, prescription refills for longer period, community based health care, limiting attendants (avoiding crowding)
  - Modify underused spaces in facilities (to limit overcrowding)
  - Health education provision, informing community on continuity of services,
  - Prioritizing vulnerable population, intervention on referral pathways
  - staffing plan like working in shifts and re-assigning staff from less busy services
  - Strategy to limit/cancel non-essential visits (**to director or delegated personnel)**
  - Strategies to ensure adequate supply for essential health service
  - Are there strategies for restoration of health care services which have been postponed
  - Were strategies on how to sustain essential health care established before the first case of COVID-19 was identified in the country or after it has been identified
  - Challenges related with mitigation strategy
  - Best practice
- What IPC measures were/are undertaken to ensure safe delivery of essential services?
  - For the providers
  - For clients
  - the challenges (acceptance of measures, supply like glove, face mask, sanitizer, water, soap)

1. Can you tell me about any kind of capacity building provided to the health facility workers to sustain essential health care service during COVID-19?

Probe

- Type of capacity building (orientation, training..)
- Who was the provider
- Was it all or group of health facility workers that were trained?

1. Was/is there a coordinating team or focal person designated to maintain essential health care services?

Probe

- Who was involved
- Role of the focal person
- Challenges identified
- Lessons learned/ best practice

1. What best practices can be identified for preventing infection among frontline healthcare workers or other patients in the healthcare setting?
2. In general, can you tell me the challenges in maintaining essential healthcare services during the COVID-19 outbreak?
3. Best practices identified in maintaining essential healthcare services during the COVID-19 outbreak?
4. Can you tell me what you think should be done to sustain essential health service during similar outbreaks for the future?
5. Anything else you want to raise in relation with essential health service provision during COVID-19?

**Health facility Key informant interview guide (NCD focal person)**

Code No. _________________________

Age_______

Sex____________

Occupation____________________

Date of Interview____________

Place of Interview__________________________________________

Time of Interview__________________________________________

1. In what ways do you think management of chronic diseases such as cardiovascular diseases, diabetes, hypertension and cancer is affected during the COVID-19 outbreak?

Probe

- What do you think was the effect related with service provision
- What do you think was the effect related with service utilization among the community
- Essential supplies for NCD

1. Can you tell me about what was done to sustain NCD health care service provision during the COVID-19 outbreak?

Probe

- What were the plans and procedures established
  - Telemedicine, prescription refills for longer period, community based health care, limiting attendants,
  - health education provision, informing community on continuity of services,
  - prioritizing vulnerable population,
  - staffing plan like working in shifts and others if mentioned
- what are the strategies developed to monitor NCD medication adherence
- What were the challenges?
- What were the best practices/lessons learned?

1. Is there follow-up mechanism for NCD patients who develop respiratory symptom?
2. What best practices can be identified for preventing infection among frontline healthcare workers or other patients in the healthcare setting?
3. In general, can you tell me the challenges in maintaining essential healthcare services during the COVID-19 outbreak?
4. Best practices identified in maintaining essential healthcare services during the COVID-19 outbreak?
5. Can you tell me what you think should be done to sustain essential health service during similar outbreaks for the future?
6. Anything else you want to raise in relation with essential health service provision during COVID-19?

**Health facility Key informant interview guide (MCH focal person)**

Code No. _________________________

Age_______

Sex____________

Occupation____________________

Date of Interview____________

Place of Interview__________________________________________

Time of Interview__________________________________________

1. In what ways do you think Maternal and child health services is affected during the COVID-19 outbreak? (ANC, delivery, PNC, FP)

Probe

- What do you think was the impact related with service provision
- What do you think was the impact related with service utilization among the community

1. Can you tell me about what was done to sustain health care services for mothers (ANC, safe delivery, PNC) during COVID-19 outbreak?

Probe

- What were the plans and procedures established (tele medication, community based service provision)
  - health education provision, informing community on continuity of services,
  - staffing plan like working in shifts and others if mentioned
- What were the challenges?
- What were the best practices/lessons?

1. Can you tell me about the mechanism to identify and reach MCH clients loss to follow-up?
2. Can you tell me about the follow-up mechanism for MCH clients who develop respiratory symptoms?
3. What best practices can be identified for preventing infection among frontline healthcare workers or other patients in the healthcare setting?
4. In general, can you tell me the challenges in maintaining essential healthcare services during the COVID-19 outbreak?
5. Best practices identified in maintaining essential healthcare services during the COVID-19 outbreak?
6. Can you tell me what you think should be done to sustain essential health service during similar outbreaks for the future?
7. Anything else you want to raise in relation with essential health service provision during COVID-19?

**Health facility Key informant interview guide (EPI focal person)**

Code No. _________________________

Age_______

Sex____________

Occupation____________________

Date of Interview____________

Place of Interview__________________________________________

Time of Interview__________________________________________

1. In what ways do you think management provision of routine immunization and supplementary immunization campaign affected during COVID-19

Probe

- What was the impact related with service provision (Any change in EPI schedule)
- What was the impact related with service utilization among the community
- Effect related with supply (unusual shortage of vaccines in the facility during COVID-19 outbreak)

1. Can you tell me about what was done/the process to sustain routine immunization during COVID-19 outbreak?

Probe

- What were the plans and procedures established
  - Telemedicine, , community based health care, limiting attendants (avoiding crowding),
  - health education provision, informing community on continuity of services,
  - staffing plan like working in shifts and others if mentioned
- What were the challenges?
- What were the best practices?

1. Can you tell me about the mechanism to record and trace children who do not come for their routine immunizations during COVID?
2. Can you tell me about the effect of COVID-19 on supplementary immunization campaigns

- What were the plans and procedures established/ measures taken to ensure routine and catch-up immunizations are not neglected during the COVID-19 outbreak?

1. What best practices can be identified for preventing infection among frontline healthcare workers or other patients in the healthcare setting?
2. In general, can you tell me the challenges in maintaining essential healthcare services during the COVID-19 outbreak?
3. Best practices identified in maintaining essential healthcare services during the COVID-19 outbreak?
4. Can you tell me what you think should be done to sustain essential health service during similar outbreaks for the future?
5. Anything else you want to raise in relation with essential health service provision during COVID-19?

**Health facility Key informant interview guide (ART focal person)**

Code No. _________________________

Age_______

Sex____________

Occupation____________________

Date of Interview____________

Place of Interview__________________________________________

Time of Interview__________________________________________

1. In what ways do you think management provision of ART service affected during COVID-19?

Probe

- What was the impact related with service provision
- What was the impact related with service utilization among the community

1. Can you tell me about what was done/ process to sustain health care services for patients on ART during the COVID-19 outbreak?

Probe

- What were the plans and procedures established
  - Telemedicine, prescription refills for longer period,
  - community based health care, health education provision, informing community on continuity of services
- What were the challenges?
- What were the best practices/lessons?

1. Is there a tracing mechanism for ART providers who developed respiratory symptom
2. Are strategies developed to monitor ART adherence (Ask what type of strategy) during COVID-19 outbreak?
3. Are strategies developed on continuity of PMTCT services (for infants who missed testing, surge strategies) during COVID-19 outbreak?
4. What best practices can be identified for preventing infection among frontline healthcare workers or other patients in the healthcare setting?
5. In general, can you tell me the challenges in maintaining essential healthcare services during the COVID-19 outbreak?
6. Best practices identified in maintaining essential healthcare services during the COVID-19 outbreak?
7. Can you tell me what you think should be done to sustain essential health service during similar outbreaks for the future?
8. Anything else you want to raise in relation with essential health service provision during COVID-19?

**Health facility Key informant interview guide (TB focal person)**

Code No. _________________________

Age_______

Sex____________

Occupation____________________

Date of Interview____________

Place of Interview__________________________________________

Time of Interview__________________________________________

1. In what ways do you think provision of TB service affected during COVID-19?

Probe to see:

- What was the impact related with service provision
- What was the impact related with service utilization among the community
- Effect on supplies for TB

1. Can you tell me about what was done/the process to sustain routine TB clinic service during COVID-19 outbreak?

Probe to see:

- What were the plans and procedures established
  - Telemedicine, prescription refills for longer period,
  - community based health care, limiting attendants (avoiding crowding),
  - health education provision, informing community on continuity of services
  - staffing plan like working in shifts and others if mentioned
- What were the challenges?
- What were the best practices?
- Is there a tracing mechanism for TB providers who developed respiratory symptom
- Are strategies developed to monitor Tb medication adherence (Ask what type of strategy) during COVID-19 outbreak?

1. What best practices can be identified for preventing infection among frontline healthcare workers or other patients in the healthcare setting?
2. In general, can you tell me the challenges in maintaining essential healthcare services during the COVID-19 outbreak?
3. Best practices identified in maintaining essential healthcare services during the COVID-19 outbreak?
4. Can you tell me what you think should be done to sustain essential health service during similar outbreaks for the future?
5. Anything else you want to raise in relation with essential health service provision during COVID-19?

**Key stakeholder key informant interview guide (FMOH, RHB)**

Code No. _________________________

Age_______

Sex____________

Profession____________________

Position___________________

Date of Interview____________

Place of Interview__________________________________________

Time of Interview__________________________________________

**Section one- planning for measures to maintain delivery of essential health care services during COVID-19**

1. Can you tell me about the coordinating structure that exist to maintain the delivery of essential health care services while freeing up capacity for the COVID-19 response?

- How is the coordinating structure organized?
- Who established this coordinating structure?
- When was this coordinating structure established
- Who are involved?
- What was the role?
- What are the key accomplishment from the coordination?

1. Is there focal person for essential health care service?

- Can you tell me about the role of this focal person?

1. Can you tell me about the strategies for maintaining essential health care services?

- Activities that has been conducted so far to maintain essential health care services?
- Mapping of health facilities including public, private and military system
- Supply chain management to ensure adequacy of supply to ensure continuity of essential health care services

1. Can you tell me how you monitor the ongoing delivery of essential health care services?

- Frequency of reporting
- Have you observed any changes in report (high/low) action taken against that?
- Tracking and monitoring of funding
- Strengthening and maintaining existing surveillances system

1. Can you tell me about activities that have been done to optimize health workforce capacity?

- Conduct rapid assessment, conduct rapid policy review of task sharing
- Requesting part-time staff to expand their hours, reassigning staffs, repurposing government and other staffs
- Can you tell me how the human resource have been identified for possible redeployment or reassignment?

**Section two- Determining which healthcare services are to be delivered to non-COVID patients along the continuum of care**

1. Can you tell the activities that have been done to determine which health services are to be delivered to non-COVID patients?

- Has the population has been stratified to assess the risk of infection by sex and health conditions
- Vulnerable groups have been considered

1. Is there a framework that has been developed that lists essential health across different setting of care and providers?

- What are the priority services?
- Can you tell how these essential services have been identified?

**Section three- Optimizing service delivery settings/platforms and coordination of providers**

1. Can you tell me how you have developed alternative mode of care to ensure sustained delivery of essential health care?

- What are these alternative mode of care? (e.g. delivering services in other location, telephone or web-based, delivering same service by different provider, spacing out the frequency of service delivery, community based delivery)
- How are these mode of care communicated with health facilities
- Support/supervision given to health facilities to ensure continuity of essential health care service
- What are the activities conducted to ensure the community are getting the necessary care through the alternative method
- Challenges related with alternative mode of care?
- Best practice related with alternative mode of care?

1. Can you tell me about the participation of other stakeholders like NGOs, Red cross and other organizations to ensure continuity of essential health care services?

**Section four- Ensuring the safety of essential health care services**

1. Can you tell me about the activities conducted to disseminate information to the public in order to guide to inform/motivate/advocate safe care seeking behaviours?

- What kind of channels were used?
- Ensure that messages are accessible to people with vision, hearing or cognitive impairment

1. What can you tell me about the standard operating procedures for facility based infection prevention and control?

- Have they been introduced or reinforced?
- Acuity-based triage is introduced at all sites
- Mechanism of isolation of patients meeting the case definition for COVID-19 are established
- Was training given for service providers on infection prevention and control?
- Clear criteria and protocols have been established for transferring patients between settings

1. In general, can you tell me the challenges in maintaining essential healthcare services during the COVID-19 outbreak?
2. Best practices identified in maintaining essential healthcare services during the COVID-19 outbreak?
3. Can you tell me what you think should be done to sustain essential health service during similar outbreaks for the future?
4. Anything else you want to raise in relation with essential health service provision during COVID-19?
