# Supplementary material for: Maintaining essential healthcare services in Addis Ababa during COVID-19: A qualitative study
Source: PLoS One. 2024 Dec 27;19(12):e0308534. doi: 10.1371/journal.pone.0308534 (PMC11676944; doi:10.1371/journal.pone.0308534)
Supplement: Attachment — Submitted filename: Review results of sustaining essential healthcare services.docx [file pone.0308534.s023.docx]

**Review results of manuscript entitled ‘sustaining essential healthcare services in Addis Ababa during COVID-19: A qualitative study’.**

I am happy to review interesting manuscript. For the better improvements and consideration I recommended to get a clear answer for the following issues before proceeding to the next process.

1. What about feasibility of this study in relation to the current status of COVID-19 and re-initiation of essential healthcare services.
2. What is the novelty of this study?
a) What is already known about the problem?
b) What is not known about the problem?
c) What is the possible implication/s for practice?
3. What does it mean by sustaining essential healthcare services?
4. What does it mean by health system’s response to maintaining essential healthcare services and sustaining essential healthcare services?
5. What about validity and reliability of the data collection tools?
6. The analysis method is not clear and strong!
7. What are the main findings & conclusion from this study? Result or hypothesis?
8. The study uses small number of participants; why?
9. What is the relationship between the objective and the result section?
10. The conclusion section is difficult to understand and very crude and vague for understanding.
11. Which citation style the authors used? Clarifying and revision may be helpful.
12. Considering English grammar and language revision is also very important.

*Author need to clarify the above points point by point to consider the next review
