# Supplementary material for: Maintaining essential healthcare services in Addis Ababa during COVID-19: A qualitative study
Source: PLoS One. 2024 Dec 27;19(12):e0308534. doi: 10.1371/journal.pone.0308534 (PMC11676944; doi:10.1371/journal.pone.0308534)
Supplement: Attachment — Submitted filename: Response to reveiwers.docx [file pone.0308534.s024.docx]

**12/20/2023**

**PLOS ONE**

**Dear Editor,**
We thank you for the opportunity to revise and resubmit this manuscript. We also appreciate constructive comments and recommendations from the reviewer. Below are our responses to the comments from the reviewer and a description of the changes we have made (texts in bullet points and italic font).

**Reviewer comment**

1. What about feasibility of this study in relation to the current status of COVID-19 and re-initiation of essential healthcare services.

- *The feasibility of this study is reflected in the fact that though COVID-19 is no longer a pandemic, we can use the findings of this study as input for inferring as a learning point to similar public health situations. Additionally, the adaptation strategies identified in this study can be used for emergency preparedness programs and decision-making. This has been described in the conclusion section of the manuscript (lines 494-496).*

2. What is the novelty of this study?
a) What is already known about the problem?
b) What is not known about the problem?
c) What is the possible implication/s for practice?

- *This study is novel because it has studied the less explored effect of COVID-19 on essential health service provision in health facilities of urban settings serving a large number of populations. The context of the study is emphasized in this study to improve the applicability of findings to similar public health situations in relation to essential healthcare services. Thus, it highlights the existing knowledge base on the effect of the pandemic on health systems as a whole and narrows in the specifics of the selected setting and phenomena for future contextualization. This is mainly highlighted in the final paragraph of the background section of the manuscript (line 83-93).*

3. What does it mean by sustaining essential healthcare services?

- *Sustaining essential healthcare services in this study is used to show the provision of uninterrupted essential healthcare services to communities. This is defined in the third paragraph of the background section on line 72-73. Note: we have used the term sustaining and maintaining essential healthcare services interchangeably in this manuscript. To avoid confusion, we have now used the word maintaining throughout.*

4. What does it mean by health system’s response to maintaining essential healthcare services and sustaining essential healthcare services?

- *The concept of the health system’s response to maintaining essential healthcare services indicates actions taken by the different levels/parts of the health sector to ensure the continued provision of health services, where the statement “maintaining essential healthcare services” holds the same meaning. However, as mentioned in the response to the above question, we have used the terms sustaining and maintaining interchangeably, which can be confusing. Hence, to avoid any confusion and to align with documents of the same concept, we have now used uniform wording, i.e., maintaining essential healthcare services. This is shown in the description of the aim of the study in the last sentence of the Background section of the manuscript.*

5. What about validity and reliability of the data collection tools?

- *We pilot-tested interview guides and made the necessary refinements before data collection to ensure the validity and reliability of data collection tool. We’ve included this under Data collection and Procedure (line 125-129).*

6. The analysis method is not clear and strong!

- *We have edited the analysis method for clarity (line 139-151).*

7. What are the main findings & conclusion from this study? Result or hypothesis?

- *The study highlights the effect of COVID-19 on the health system and the health sector's action to ensure the continued provision of essential health services during the pandemic's initial phase. The main findings of this study are summarized in the first paragraph of the discussion. (line 430-442).*

8. The study uses small number of participants; why?

- *We determined the sample size based on the saturation of information. We have clarified this in (line 117-118).*

9. What is the relationship between the objective and the result section?

- *This study was conducted with the aim of exploring the actions taken by the health sector to ensure sustained provision of essential healthcare services during the COVID-19 pandemic. With this study, we were able to highlight the impact of COVID-19 on service utilization and provision. The result also highlighted key adaptation strategies implemented by the health sector to sustain the provision of essential healthcare services and the challenges encountered. These key findings have been reported in the result section and were discussed accordingly.*

10. The conclusion section is difficult to understand and very crude and vague for understanding.

- *We have edited the conclusion section for clarity (line 495-505).*

11. Which citation style the authors used? Clarifying and revision may be helpful.

- *We have used the Vancouver style for citation. Revision to the reference section has been made accordingly.*

12. Considering English grammar and language revision is also very important.

- *We have made edits to the manuscript for English grammar and language. All authors reviewed and edited the manuscript.*
