# Supplementary material for: Maintaining essential healthcare services in Addis Ababa during COVID-19: A qualitative study
Source: PLoS One. 2024 Dec 27;19(12):e0308534. doi: 10.1371/journal.pone.0308534 (PMC11676944; doi:10.1371/journal.pone.0308534)
Supplement: Attachment — Submitted filename: Response to reveiwers.docx [file pone.0308534.s025.docx]

**5/25/2024**

**PLOS ONE**

**Dear Editor,**
We thank you for the opportunity to revise and resubmit this manuscript. We also appreciate constructive comments and recommendations from the reviewer. Below are our responses to the comments from the reviewer and a description of the changes we have made (texts in bullet points and italic font).

**Editor’s comment**

Domain 1: Research Team and Reflexivity
- Interviewer/Facilitator: Interviews were conducted with participants, but details regarding the credentials, occupation, gender, experience, training, and relationship establishment of the interviewers/facilitators remain undisclosed.

*- Thank you for your comments. We have included the details of the interviewers in the methods section (line 143-147).*

Domain 2: Study Design
- Theoretical Framework: The manuscript lacks explicit reference to a theoretical framework, which is pivotal for contextualizing the study.

*- Thank you for the comments. We have used an exploratory study design to guide this study. We have now included the study design in the method section under study design and setting section (line 97-100).*

- Participant Selection: Purposive sampling was employed, yet details regarding the method of approach, sample size, non-participation, setting of data collection, presence of non-participants, and description of the sample are absent.

*- Thank you for the comments. We have included the method of approach, sample size, non-participation, setting of data collection, presence of non-participants, and the study size in the method section under Sample size and study participants (line 114-126). Furthermore, the sample description is now included in the first part of the result (line 199-202).*

Domain 3: Analysis and Findings

- Interview Guide: The development and pilot testing of interview guides are not addressed, which is crucial for ensuring data collection validity.

*- The aspects of interview guide development, components of the guide, pilot testing and changes as a result of pilot testing are addressed in the “Data collection tool and procedure” sections (line 129-138)*
- Data Analysis: Although themes were derived from the data, information regarding the number of data coders, description of the coding tree, software used for analysis, and participant checking is notably absent.

*- Thank you for your comments. We have addressed issues of coding in the methods and materials section under data analysis and we have provided description of the coding tree at the beginning of the results section (line 203-108). The software used for analysis is also indicated in the analysis section (line 165). We have now included details of rigor in the Methods and materials section under rigor (line 168-184).*

Reporting

- Quotations Presented: Participant quotations are utilized to illustrate themes/findings, but consistency between data presented and findings, clarity of major and minor themes, and discussion of diverse cases warrant improvement for enhanced clarity and transparency.

- *Thank you for the important insight. We have indicated the themes and sub-themes to show the major and minor themes and their relationship, we have also included the sources of the quotations to create more clarity and transparency.*

**Reviewers' comments**

I have a few concern and questions on this work:

1. Why are the authors interested to employ only key informant interviews? Why are they interested in interviewing only "highly knowlesgeable" respondents? What does highly knowledgeable even mean? For example let me just use one of your findings to explain my concern. It was found out that discontinuance of service utilization (such as ART follow_up). Were you thinking that the drivers and means of mitigation of such discontinuece is most understood and addressed through the recomendations and ideas of highly knowledgeable health system workers alone? What do you think was the role of discussing with communities or beneficiaries (through indepth interviews and FGDs)? Do not you think missing relevant ideas from such population would have benefited the study? Moreover, do not you think that mitigations and maintainace of essential services are collective and participatory responses whereby community engagement should be manifested in? Hence, I see some limitation on the study design and methods which should be honestly reported in the study.

- *Thank you for the valuable comment and insight. We accept that this study would have been more comprehensive if the community’s perspective had been included. Thus, we have put this as a limitation of this study (line 539-540).*
- *With the word “knowledgeable”, we were trying to indicate that the participants possibly had comprehensive information about the effect of COVID-19 and the mitigation strategies and/ or the health system response to sustain the provision of essential healthcare services in their respective departments. Thus, we have removed the term knowledgeable to avoid ambiguity and replaced it with the meaning it portrays (line 114-118).*

2. Can you please give a separate section in the methods for "rigor"? Wherein you discuss the main dimensions of trusthworthineass of the study and how you implemented them: crediblity, depedabality, transferrablity, and conformablity
Thank you for the great job

*- We have now created a separate rigor section in the methods and materials and included details about activities conducted to ensure trustworthiness (line 168-184)*
